# Study on the influence of levels of physical activity and socio-economic conditions on body mass index of adolescents

**DOI:** 10.1093/inthealth/ihae083

**Published:** 2024-11-21

**Authors:** Li Liu, Yongsen Liu, Tingran Zhang, Jiong Luo

**Affiliations:** Research Centre for Exercise Detoxification, College of Physical Education, Southwest University, Chongqing, China 400715; Research Centre for Exercise Detoxification, College of Physical Education, Southwest University, Chongqing, China 400715; Research Centre for Exercise Detoxification, College of Physical Education, Southwest University, Chongqing, China 400715; Research Centre for Exercise Detoxification, College of Physical Education, Southwest University, Chongqing, China 400715

**Keywords:** adolescent obesity, body mass index, multiple logistic regression, physical activity level, socio-economic status

## Abstract

**Background:**

We explored the relationship between adolescent physical activity levels, socio-economic conditions and body mass index (BMI) in order to gain a deeper understanding of the relevant factors affecting adolescent obesity.

**Methods:**

A stratified random sampling method was used to conduct a questionnaire survey of middle school students in the Chengdu–Chongqing Economic Zone. Multiple linear and logistic regression analysis methods were used to statistically analyse the data obtained.

**Results:**

The level of moderate to vigorous physical activity (MVPA) not only significantly reduces the incidence of obesity in adolescents, it also has a positive effect on avoiding underweight in adolescents. The impact of a father's BMI on a son's weight is higher than that of a daughter, while the impact of a mother's BMI on a child's weight is the opposite. High monthly income has a positive effect on reducing the BMI of male and female adolescents, but full-time working mothers actually increase the risk of obesity in their children. Teenagers who have exercise habits or view exercise as a form of enjoyment have a significantly reduced risk of obesity.

**Conclusions:**

The level of MVPA and exercise habits are important factors in inhibiting the development of obesity in adolescent students.

## Introduction

In the past few decades, the global prevalence of obesity among children and adolescents has significantly increased, with an increasing trend in both developed and developing countries.^1,2^ Research shows that the global prevalence of obesity among children and adolescents ages 5–19 y has increased from 0.7% in 1975 to 5.6% in 2016 and from 0.9% in 1975 to 7.8% in 2016, respectively.^3^ According to World Health Organization (WHO) estimates, by 2025 there will be >70 million overweight or obese children worldwide. Therefore, in the 2013–2020 Global Strategic Action Plan for the Prevention and Control of Noncommunicable Diseases, the WHO proposed the goal of stopping the growth of global childhood and adolescent obesity rates by 2025. In Asia, the regions with the highest absolute increase in the number of obese children and adolescents are East Asia, the Middle East, North Africa, South Asia and high-income English-speaking regions. The prevalence of childhood obesity is increasing in almost all countries, with low- and middle-income countries experiencing the fastest increase.^4,5^ China has become one of the countries with the highest number of obese children. As early as 2015, the number of obese children and adolescents ages 5–19 y in China ranked first in the world, but there were significant regional differences.^6–8^

Obesity is a multifaceted chronic disease that involves elements such as environment, genes, metabolism and behavioural patterns. A literature review indicates that the causes of obesity are complex and diverse, including social and cultural factors, lifestyle, physical activity, dietary habits and changes in the family environment. It is also pointed out that the popularity of fast food may be related to the increase in obesity in children and adolescents.^9–11^ Research has found that children who are overweight or obese during childhood or adolescence are more likely to become overweight or obese in adulthood compared with normal weight children.^12^ About three-fourths of obese adolescents may be potential patients with cardiovascular diseases, diabetes and other chronic diseases in the future.^13^ Further research has found that engaging in physical activity during childhood and adolescence reduces the risk of obesity in adulthood and has a positive effect on blood pressure and emotions.^14^ Regular exercise can also effectively improve the occurrence of type 2 diabetes and improve the quality of life of type 2 diabetes patients.^15^ Specifically, moderate-intensity physical activity can effectively improve short- and long-term cardiopulmonary function in obese children and adolescents,^16^ while children and adolescents lacking sufficient physical activity levels are more likely to continue engaging in insufficient physical activity levels in adulthood.^17^

In the study of the relationship between socio-economic status, environmental factors, physical activity and obesity, it was found that socio-economic status is positively correlated with obesity.^18,19^ Some studies have also found that there is no significant relationship between socio-economic status and obesity.^20^ In Asia, most studies have found a negative correlation between household income and overweight and obesity rates, with a trend of higher income leading to lower rates of overweight and obesity. These research findings roughly echo international research findings. In a study of the relationship between socio-economic status and physical activity, it was found that parents can influence their children's physical activity behaviour by controlling their time and resource structures.^21,22^ The higher the social and economic status, the greater the demand for sports and the higher the frequency of spontaneous physical and mental activities. Moreover, those with wealthier families have higher levels of physical, psychological and life satisfaction.^21,23^ Of course, some studies have found the opposite, suggesting that adolescents from low socio-economic status families engage in more physical activities, while those from high socio-economic status families have higher social capital and are more likely to have high expectations for their children, hoping that they can enter higher-ranked schools. Therefore, they tend to convert their economic capital into tutoring, resulting in a significantly higher proportion of children participating in subject tutoring, which in turn reduces the time adolescents can engage in physical activities.^24,25^ In the study of fitness environment and physical condition, it was found that high-income families have higher consumption ability than low-income families and have more resource choices and environmental conditions. Their socio-economic status is significantly negatively correlated with overweight and obesity.^26,27^ Adolescents living in a better walking environment have more opportunities for physical activity by walking to school.^28^

Previous literature has mostly emphasized the negative effects of overweight and obesity, and a healthy environment and physical activity are policy issues that countries around the world need to continue to pay attention to.^29^ The causal relationship between sociodemographic variables and health environment characteristics, as well as adolescent overweight and obesity, is worth further exploration. Moreover, how is physical activity included in this research process, especially the impact of walking time on the weight status of children and adolescents?

Based on this, this study used the International Physical Activity Questionnaire (IPAQ) short version to calculate the physical activity level of adolescents. Based on the social dynamics of obesity (SDO) as the theoretical basis, a survey was conducted on adolescents in the Chengdu–Chongqing Economic Zone (CCEZ) in western China to explore the impact of their physical activity level and economic and social conditions on their weight status. A multiple logistic regression model was used, with a reference category of adolescents with normal weight as the baseline, to identify the causal relationship between adolescent weight status and various possible factors. This provides important reference suggestions for reducing the prevalence of adolescent obesity, improving national health conditions and reducing social medical costs.

## Methods

### Respondents

This study focuses on middle school students (grades 10–12) in the CCEZ. We ranked the economic development status of each district and then randomly selected one school in each district. G-power version 3.1 software was used to estimate the sample size, with a type Ⅰ error α value of 0.05, a statistical test power of 0.9 and an effective size of 0.5. Based on this, a sample size of 3500 was determined, and considering a 20% sample loss rate, 4200 questionnaires were distributed. The members of the research group explained the questionnaire content and the students completed the questionnaire. The members of the research group provided immediate explanations and assistance if there were any questions.

The questionnaire collection period was from May 2023 to August 2023. A total of 4167 questionnaires were collected (including 1345, 1423 and 1399 for grades 1, 2 and 3, respectively). A total of and 4012 valid questionnaires were collected. The sampling rate is directly proportional to the size of the population, i.e. the number of samples in each district is directly proportional to the total population of students in each district (see Table 1).

**Table 1.** Distribution of formal survey samples.

		Gender, n		Grade, %			
Area	Sample distribution, n	Male	Female	High school 1	High school 2	High school 3	Total, N
Chengdu	Key schools, 1050	550	500	400	335	315	1050
	Ordinary schools, 1150	600	550	410	385	355	1150
Chongqing	Key schools, 900	460	440	305	300	295	900
	Ordinary schools, 912	490	422	310	312	290	912
Total		2100	1912	1425	1332	1255	4012

### Questionnaire design

Using a cross-sectional survey analysis model, the questionnaire included two parts: personal basic information and physical activity levels. The survey content of the personal basic data included students’ gender, age, height, weight, parents’ height and weight, parents’ working status (whether they work full-time or not), the number of brothers and sisters, family cohabiting members, family monthly income, tutoring situation, sports purpose, personal pocket money, etc. Physical activity level was evaluated based on the short version of the International Physical Activity Questionnaire (IPAQ). Students were asked to recall the frequency and duration of intense (8 metabolic equivalent of tasks [METs]), moderate (4 METs) and mild (3.3 METs) leisure physical activities over the past 7 d, excluding physical activities such as physical education classes, household chores and work. The intensity of each physical activity level (METs) was multiplied by the amount of time engaged to convert it into weekly physical activity levels (METs min/week). After being translated into Chinese, this questionnaire was confirmed to have sufficient reliability and validity (retest reliability r=0.84; internal consistency test, Cronbach’s α=0.87) before the formal questionnaire.

### Definitions of variables

#### Obesity

The dependent variable (Y) of this study is the weight status of adolescents. Based on the weight and height data provided by the survey subjects, the body mass index (BMI) was determined (BMI=weight [kg]/height [m^2^]). According to the definition of obesity established by the Chinese adolescent physical and health standards,^30^ adolescent weight is divided into four categories: underweight (BMI <18.5), normal (BMI ≥18.5–<24.9), overweight (≥24.9–<28) and obese (BMI ≥28). The investigated population was divided into groups based on their weight status, with the normal weight group as the reference baseline. The dependent variable Y was set as follows: underweight (Y=1), normal weight (Y=2), overweight (Y=3) and obese (Y=4).

#### Weekly physical activity levels (METs min/week)

The weekly physical activity level was evaluated based on the physical activity intensity values (METs) provided by the IPAQ. It uses vigorous exercise (8 METs), moderate exercise (4 METs) and walking for >10 min (3.3 METs) and then calculates METs based on the data provided by the student. The weekly physical activity level is obtained by multiplying the physical activity intensity values (METs) by the exercise time to obtain the weekly physical activity level of the subjects. Referring to the methods of relevant scholars,^31,32^ the METs of adolescents ranged from 0 to 850, with the first group (represented by SD1) representing the low-exercise group; from 851 to 1500 (represented by SD2), indicating that the exercise volume meets the standard group; and >1501 (represented by SD3), representing the high-exercise group. It was expected that there is a negative relationship between physical activity level and adolescent weight status.

#### Other variables

##### Age

Age was investigated to determine whether it is related to the weight status of adolescents and explores whether there is a linear relationship between age and weight status.

##### Father's BMI

Using the father's height and weight data, we calculated the BMI of the respondents’ fathers to understand whether there is a relationship between the fathers’ BMI and adolescent BMI.

##### Mother’s BMI

Using the mother's height and weight data, we calculated the BMI of the respondents’ mothers to understand whether there is a relationship between the mothers’ BMI and adolescent BMI.

##### Extracurricular tutoring

We looked at the tutoring behaviour of adolescents to determine if it is related to their weight status, as students who have tutoring may spend less time exercising and have increased sedentary time. Therefore, it is expected that tutoring will have a positive relationship with the weight status of adolescents (baseline group=no tutoring).

##### Father's work status

The survey investigated whether the father of the survey subject had a full-time job in the past year and whether the father's work had an impact on the weight status of the adolescent (baseline group=father did not have a full-time job).

##### Mother's work status

The survey investigated whether the mother of the survey subject had a full-time job in the past year to determine whether the mother's work had an impact on the weight status of the adolescent (baseline group=mother did not have a full-time job).

##### Brothers and sisters

We wanted to determine if there is a relationship between having brother(s)/sister(s) and the adolescent’s weight (baseline group=no brothers and sisters).

##### Co-residents

There were four options: (A) living with parents, (B) living with parents and grandparents, (C) living only with grandparents and (D) other. To explore whether living with parents affects the weight of adolescents, if the survey subject selected (A) or (B), the dummy variable was set to 1, indicating that they live with their parents. If the respondent selected (C) or (D), the dummy variable was set to 0, indicating that they do not live with their parents (baseline).

##### Monthly household income

There were two options: (A) ≥15 000 yuan/month and (B) <15 000 yuan/month. If (A) is selected, the dummy variable is set to 1 (high-income family). If (B) is selected, the dummy variable is set to 0 (baseline group=low-income family).

##### Sufficiency of pocket money

The survey asked whether an individual's current pocket money is sufficient, with a total of four options: (A) quite sufficient and surplus, (B) just enough, (C) a bit insufficient and (D) very insufficient. If option (A) or B) is selected, the individual believes that his/her pocket money is sufficient. If option (C) or (D) is selected, the individual believes that his/her pocket money is not enough (baseline group=personal pocket money is not enough).

##### Purpose of sports

The respondents were asked about the purpose of sports? The options included lifestyle habits, wanting to be with family or friends, having fun and passing the time. If checked, set to 1, otherwise set to 0 (baseline group=0 ethnic group).

### Mathematical statistics

We classified and encoded the collected data based on variable characteristics and established a database. SPSS 21.0 (IBM, Armonk, NY, USA) was used for processing, including descriptive analysis, correlation analysis and multiple linear and logistic regression analysis. The significance level of all indicators was set at α=0.05.

The reason for choosing a combination of linear regression and logistic regression is that the dependent variable (Y) of this study—adolescent BMI—is a continuous measurement variable, so multiple linear regression can only be considered. However, multiple linear regression theory requires it to follow the assumption of normal distribution and equal lines (linear, independent, normal, equal variance) and the principle requires the independent variable (X) to be a continuous variable, which is sometimes difficult to ensure in practical problem exploration. In addition, in exploring the impact of the independent variable (X) on the dependent variable (Y) through multiple linear regression, only approximate effects can be obtained and relatively accurate odds ratios (ORs) cannot be obtained. The dependent variable (Y) in logistic regression is a categorical variable, while the predictor variable (independent variable) can be either continuous or categorical, and there is no requirement for the variable to satisfy a normal distribution. In addition, logistic regression can obtain ORs and accurately assess the influence of the independent variable (X).

The commonly used model for the OR is, let the dependent variable Y have j categories, with probabilities of P_1_, P_2_ and P_j_ and satisfying P_1_+P_2_+…P_j_=1. Based on these probabilities, n independent observation objects are assigned to their respective categories and the distribution of observation objects in j categories follows a polynomial distribution. When J=2, the dependent variable Y of the polynomial distribution becomes a binary logistic regression. The independent variable is denoted as X_jk_ (k=1, 2, 3, …, m). The multimodal logit model for unordered classification can be expressed as


\begin{eqnarray*}
\ln \left( {\frac{{{{{\hat{p}}}_j}}}{{{{{\hat{p}}}_J}}}} \right) = {{\alpha }_j} + {{b}_{j1}}{{X}_1} + \ldots {{b}_{jk}}{{X}_k} + \ldots + {{b}_{jp}}{{X}_{ip}};\quad j = 1, \ldots J - 1
\end{eqnarray*}

This equation is based on the last category (J) as the baseline (other categories can also be selected as the baseline) and a regression model is established between each reaction category j and the baseline category J. Therefore, this model is also known as the baseline classification logistic model. This model requires simultaneous estimation of (J−1) binomial classification logistic models, with a wide range of applications and high flexibility, also known as the generalized logistic model. Generally, for a reference baseline (OR=1), if OR is >1, it indicates that the influencing factor is a favourable factor for Y; otherwise, it is not.

## Results

### Relationship between social background variables and physical activity levels

The proportion of high, medium and low physical activity levels among adolescents was 8.7%, 41.5% and 49.9%, respectively, and there were significant gender differences (χ^2^=166.89, p=0.001) and school characteristics differences (χ^2^=62.92, p=0.01). The proportion of moderate to vigorous physical activity (MVPA) levels among males was significantly higher than that among females, and the proportion of high physical activity levels among adolescents in regular schools was significantly higher than that in key schools. The proportion of people with low physical activity levels in key schools was significantly higher than that in ordinary schools.

A total of 65.8% of teenagers participate in extracurricular tutoring, while only 34.2% of students do not. Through contingency analysis, it was found that there were no gender or regional differences in the participation of students in extracurricular tutoring, but there were significant differences in school characteristics (χ^2^=597.37, p=0.001). The proportion of students participating in tutoring in key schools was significantly higher than that in ordinary middle schools.

The main purpose of adolescent sports is to pass the time (34.4%), followed by for fun (30.5%), with only 25.6% of adolescents considering sports as a lifestyle habit. There are significant gender and school characteristics differences in the purpose of adolescent sports (χ^2^=100.99, p=0.001; χ^2^=83.12, p=0.001), among which males and key high school adolescents view sports more as a lifestyle habit. However, women and adolescents in regular high schools tend to see sports as for fun, as well as wanting to play with family (or friends) (see Table 2).

**Table 2.** Correlation between the physical activity level of adolescents and their socio-economic conditions (N=4012).

Variables		Gender, n (%)			Region feature, n (%)			School characteristics, n (%)		
		Male	Female	Total	Chengdu	Chongqing	Total	Key schools	Ordinary schools	Total
Physical activity level	High	273 (13.0)	75 (3.9)	348 (8.7)	173 (8.8)	175 (8.6)	348 (8.7)	104 (5.3)	244 (12.0)	348 (8.7)
	Medium	949 (45.2)	715 (37.4)	1664 (41.5)	824 (41.6)	840 (41.2)	1664 (41.5)	815 (41.2)	849 (41.7)	1664 (41.5)
	Low	878 (41.8)	1122 (54.4)	2000 (49.9)	978 (41.8)	1022 (49.6)	2000 (50.2)	1058 (53.5)	942 (46.3)	2000 (49.9)
	χ^2^=166.89, p=0.001				χ^2^=0.134, p=0.935			χ^2^=62.92, p=0.01		
Supplementary education situation	Ⅰ	1421(67.1%)	1219(64.4%)	2640(65.8%)	1405(67.0%)	1235(64.5%)	2640(65.8%)	1781(82.8%)	859(46.1%)	2640(65.8%)
	Ⅱ	698(32.9%)	674(35.6%)	1372(34.2%)	691(33.0%)	681(35.5%)	1372(34.2%)	369(17.2%)	1003(53.9%)	1372(34.2%)
	χ^2^=3.156, p=0.076				χ^2^=2.950, p=0.086			χ^2^=597.37, p=0.001		
Work status of parents	Ⅲ	1387(69.8%)	1482(73.2%)	2869(71.5%)	1397(69.6%)	1472(73.5%)	2869(71.5%)	1500(71.8%)	1369(71.2%)	2869(71.5%)
	Ⅳ	601(30.2%)	542(26.8%)	1143(28.5%)	611(30.4%)	532(26.5%)	1143(28.5%)	588(28.2%)	555(28.8%)	1143(28.5%)
	χ^2^=3.85, p=0.061				χ^2^=3.05, p=0.059			χ^2^=0.231, p=0.631		
Sport purpose	Ⅴ	601(27.9%)	623(33.5%)	1224(30.5%)	613(29.9%)	611(31.2%)	1224(30.5%)	630(28.3%)	594(33.2%)	1224(30.5%)
	Ⅵ	681(31.6%)	346(18.6%)	1027(25.6%)	536(26.1%)	491(25.1%)	1027(25.6%)	688(30.9%)	339(19.0%)	1027(25.6%)
	Ⅶ	715(33.2%)	665(35.8%)	1380(34.4%)	725(35.3%)	655(33.4%)	1380(34.4%)	735(33.0%)	645(36.1%)	1380(34.4%)
	Ⅷ	158(7.3%)	223(12.0%)	381(9.5%)	178(8.7%)	203(10.4%)	381(9.5%)	171(7.7%)	210(11.7%)	381(9.5%)
	χ^2^=100.99, p=0.001				χ^2^=5.059, p=0.168			χ^2^=83.12, p=0.001		

Classification meaning: I, participate in tutoring; II, did not participate in tutoring; Ⅲ, both parents are work full time; IV, parents do not work full time; V: for fun; VI: living habits; VII: pass the time; VIII: play with family (or friends).

### Multiple linear regression model analysis of factors affecting adolescent BMI

Overall, the age of adolescents (β=0.0411, p=0.001), want to play with family (friends) (β=0.0316, p=0.001), gender (β=0.0255, p=0.001), mother's BMI (β=0.0157, p=0.001) and father's BMI (β=0.0127, p=0.001) significantly increases BMI in adolescents. The size of the value indicates that adolescent age has the greatest influence, followed by wanting to play with family (friends), gender, mother's BMI and father's BMI. The monthly income of the family (β=−0.0257, p=0.001) and for fun (β=−0.0366, p=0.001) significantly negatively affects the BMI of adolescents, with the impact of exercise goals being greater for fun than for family monthly income.

After excluding the influence of gender factors, the work status of father (β=−0.0402, p=0.01) and mother (β=−0.0289, p=0.05) significantly negatively affects the BMI of daughters, with fathers having a greater impact than mothers. In addition, there are significant gender differences in the impact of exercise purpose on adolescent BMI. Girls who consider exercise as their lifestyle habit also have significant gender differences (β=−0.0422, p=0.01), significantly negatively affecting their BMI. Boys who view exercise as wanting to play with family (friends) (β=0.0399, p=0.05) significantly positively affect their BMI, while boys who choose for fun as their exercise goal (β=−0.0607, p=0.001) significantly negatively affect their BMI (see Table 3).

**Table 3.** Linear relationship analysis of factors influencing adolescent BMI.

	Overall			Male			Female		
Factors	β coefficient	p-Value	Significance level	β coefficient	p-Value	Significance level	β coefficient	p-Value	Significance level
Moderate activity level	−0.0189	0.271		−0.0249	0.396		−0.0136	0.497	
High activity level	−0.0091	0.533		−0.0134	0.555		−0.0081	0.602	
Gender	0.0411	0.001	0.001						
Age	0.0255	0.000	0.001	0.0332	0.000	0.001	0.0179	0.008	0.01
Father BMI	0.0127	0.000	0.001	0.0143	0.000	0.001	0.0105	0.000	0.001
Mother BMI	0.0157	0.000	0.001	0.0088	0.007	0.01	0.0194	0.000	0.001
Private Supplementary Tutoring	0.0054	0.615		0.0077	0.716		0.0025	0.877	
Father's Work Status	0.1401	0.529		0.0091	0.772		−0.0402	0.008	0.01
Mother's Work Status	0.0155	0.612		0.0044	0.846		−0.0289	0.041	0.05
Sibling	−0.0328	0.123		−0.0358	0.305		−0.0150	0.549	
Co residents	−0.0108	0.625		−0.0058	0.865		−0.0128	0.641	
Monthly household income	−0.0257	0.090	0.001	−0.0224	0.426	0.001	−0.0261	0.079	0.001
Adequacy of pocket money	0.0058	0.673		0.0133	0.515		−0.0053	0.704	
habits and customs	−0.226	0.671		−0.0073	0.729		−0.0422	0.026	0.01
Want to play with family (friends)	0.0316	0.021	0.01	0.0399	0.046	0.05	0.0213	0.186	
Fun/ interest	−0.0366	0.006	0.001	−0.0507	0.005	0.001	−0.0128	0.413	
pass the time	−0.0009	0.940		−0.0009	0.965		−0.0083	0.599	
Intercept	2.4015	0.000	0.001	2.3415	0.000	0.001	2.3617	0.000	0.001
F/P	F=16.55, p=0.001			F=8.56, p=0.01			F=12.69, p=0.001		
R^2^	0.1128			0.1095			0.1788		

### Multivariate logistic regression analysis of factors affecting adolescent BMI

The level of physical activity significantly affects the BMI of adolescents. Overall, if the level of physical activity changes from low to medium or high, the incidence of obesity in adolescents will decrease by 0.56 times and 0.68 times, respectively. Excluding the influence of gender, if the physical activity level of male adolescents changes from low to moderate, their obesity rate decreases to 0.43 times the original. If the physical activity level of female adolescents is increased from low to high, their obesity rate decreases to 0.33 times the original.

The BMI of parents significantly affects the BMI of adolescents. Overall, for every 1 unit increase in the BMI of parents, the incidence of underweight in adolescents decreases to 0.85 times that of the father and 0.87 times that of the mother. However, for every 1 unit increase in the BMI of fathers, the incidence of obesity in adolescents increases to 1.17 times the original level. Excluding the influence of gender, for every 1 unit increase in the father's BMI, the incidence of obesity in men increases by 1.28 times and the overweight rate in women increases by 1.16 times, while for every 1 unit increase in the mother's BMI, the obesity rate in women increases by 1.21 times but it has no effect on men.

Overall, the father's work status has no effect on adolescent BMI, but the mother's work status is the opposite. When the mother's work changes from part time to full time, the adolescent obesity rate will increase to 1.60 times the original. When the father's work status changes from part time to full time, the incidence of obesity in female adolescents decreases by 0.28 times, but when the mother's work status changes from part time to full time, the incidence of obesity in male adolescents increases to 2.77 times.

Family monthly income significantly affects the BMI of adolescents. Overall, when family monthly income increases from low to high, the incidence of obesity in adolescents decreases to 0.58 times the original level. Excluding gender factors, the incidence of overweight in male adolescents decreases to 0.82 times and the incidence of obesity in female adolescents decreases to 0.34 times the original level.

Exercise habits significantly affect the BMI of adolescents. Overall, those who view exercise as a lifestyle habit have a 0.81 times lower incidence of underweight, while the incidence of obesity in women is 0.26 times higher. If exercise is only considered as a way to play with family (friends), the incidence of obesity in female adolescents increases by 2.62 times. If sports are treated as fun, the incidence of overweight in male adolescents decreases by 0.48 times, while the incidence of underweight in women decreases by 0.78 times (see Table 4).

**Table 4.** Multivariate logistic regression analysis statistical table of factors affecting adolescent BMI.

Variables		Overall (OR)			Male (OR)			Female (OR)		
		Underweight	Overweight	Obese	Underweight	Overweight	Obese	Underweight	Overweight	Obese
Physical activity level	Low	–	–	–	–	–	–	–	–	–
	Moderate	0.66	0.90	0.56**	0.53*	0.82	0.43*	0.80	1.16	0.48
	High	0.73	0.67	0.68**	0.77	0.70	0.68	0.67	0.63	0.33***
Age		1.18*	1.06	1.01	1.01	0.87	1.16	1.44***	1.45**	0.72
Father BMI		0.85***	1.06	1.17***	0.88***	1.02	1.28*	0.86***	1.16**	1.12
Mother BMI		0.87***	1.01	1.01	0.91**	1.00	1.00	0.87***	1.07	1.21***
Extracurricular tutoring	No	–	–	–	–	–	–	–	–	–
	Yes	1.06	1.07	0.86	1.06	1.17	0.99	1.06	0.96	0.89
Father works full time	No	–	–	–	–	–	–	–	–	–
	Yes	0.77	0.72	0.73	0.53	1.17	0.92	1.62	0.98	0.28*
Mother works full time	No	–	–	–	–	–	–	–	–	–
	Yes	1.15	0.76	1.60*	1.28	0.75	1.30	0.99	0.88	2.77**
Sibling(s)	No	–	–	–	–	–	–	–	–	–
	Yes	1.24	0.73	0.68	0.96	0.77	0.58	1.52	0.67	1.08
Co-residents	No	–	–	–	–	–	–	–	–	–
	Yes	1.62	0.91	0.89	0.86	0.78	0.64	1.05	1.03	1.42
Monthly household income	Low	–	–	–	–	–	–	–	–	–
	High	1.23	1.29	0.58***	1.06	0.82*	0.62	1.51	0.86	0.34***
Adequacy of pocket money	No	–	–	–	–	–	–	–	–	–
	Yes	0.89	1.22	0.77	0.73	1.30	0.70	1.08	0.99	0.76
Habits and customs	No	–	–	–	–	–	–	–	–	–
	Yes	0.81**	1.13	0.73	1.53	1.30	1.33	1.57	1.05	0.26***
Want to play with family (friends)	No	–	–	–	–	–	–	–	–	–
	Yes	0.88	1.01	0.89	0.67	0.88	1.33	1.08	1.01	2.62**
Fun	No	–	–	–	–	–	–	–	–	–
	Yes	0.60**	0.69*	0.82	1.08	0.48***	0.60	0.78***	1.21	1.08
Pass the time	No	–	–	–	–	–	–	–	–	–
	Yes	0.84	1.04	0.89	0.81	0.98	0.86	0.96	1.11	0.88

*p=0.05, **p=0.01 and ***p=0.001.

The ORs for BMI, overweight and obesity were all based on the normal weight group as the baseline: OR >1, favourable factors; OR <1, unfavourable factors.

## Discussion

Overweight and obesity has become a global public health issue that urgently needs to be addressed. According to the report on the nutrition and chronic disease status of Chinese residents (2020), the overweight/obesity rate of children and adolescents in China is 19% (China, EB/OL, 2021).^5^ Research has found a high correlation between low levels of physical activity and cardiovascular disease status. Individuals with a low level of physical activity are often at risk of developing cardiovascular disease and autonomic dysfunction. Compared with the general population, those who engage in long-term exercise have lower body fat rates and higher lean weight,^33^ while those who maintain MVPA for a long time have lower BMI, less work pressure and shorter sleep time.^34^ This study conducted a survey of middle school students in the CCEZ. From the overall sample size, the proportion of high, medium and low physical activity levels among adolescents was 8.7%, 41.5% and 49.9%, respectively. Among these, the proportion of low physical activity levels in key schools was 53.5% and the proportion of low physical activity levels among women was 54.4%. These data are relatively superior to the overall data reported by global youth.^35^ According to relevant reports, about 81% of teenagers ages 11–17 y worldwide have insufficient physical activity levels and there is a significant gender difference in the proportion of MVPA exercise.^36^ The proportion of MVPA among female teenagers (20%) is much lower than that of males,^37^ which may have a negative impact on their physical health.

Physical activity level, sedentary behaviour and sleep are the three main activity behaviours of adolescents throughout the day. If one activity time increases, it will inevitably lead to a decrease in another activity. The Canadian and Australian Sports Physiology Association has organized experts from various fields to develop and issue 24-h activity guidelines for children and adolescents, which propose that the daily MVPA time for children and adolescents should be >60 min.^38,39^ This study found that physical activity levels have a significant negative impact on the likelihood of obesity in adolescents. Compared with adolescents who do not reach the minimum weekly physical activity level, adolescents with moderate and high physical activity levels are only 0.56 times and 0.68 times more likely to become obese, respectively. Additionally, males with moderate physical activity levels are only 0.42 times more likely to have a low BMI. It can be seen that exercise is not only beneficial for obese boys, but also for boys who are underweight, because extreme overweight or underweight has significant adverse consequences on physical health, which means that there is still a risk of mortality similar to that of overweight in underweight cases.^40^ However, previous literature has focused more on exploring the impact of exercise on weight in overweight and obese populations, so this finding is worthy of attention. Can exercise help subjects reduce the likelihood of underweight and further reduce the risk of mortality? This study found that although it confirms the research conclusions of many scholars both domestically and internationally,^41–43^ it is also inconsistent with a few studies on MVPA.^44,45^ i.e. the physical activity level of adolescents is not related to overweight/obesity.

Since adolescent obesity is not only influenced by physical activity, the influence of sociodemographic variables and construction environment characteristics on overweight and obesity cannot be ignored. Many researchers often use walking time as a variable to measure physical activity levels, and have found that total walking time significantly negatively affects the probability of obesity, but it does not have a significant impact on overweight populations.^46^ However, physical activity behaviour can be divided into mild, moderate and vigorous activities, and the level of physical activity for each activity is different. For example, walking is classified as mild physical activity, which consumes 3.3 METs/min. Therefore, it is not very accurate to evaluate an individual's physical activity level based solely on walking time. This study drew on the shortcomings of previous research methods and used the IPAQ short version to accurately calculate the physical activity level of each adolescent in the past week, exploring its impact on weight status. The results showed that physical activity level had a significant impact on obese populations and had almost no effect on overweight populations.

There is controversy among scholars regarding the relationship between MVPA and adolescent overweight/obesity in research, which may be attributed to many reasons, but can be summarized in two main aspects: first, it may be inconsistent with the physical activity level measurement tools used by different students, and there are original and simplified versions of the IPAQ. Second, due to the fact that the vast majority of studies are cross-sectional surveys, when exploring the correlation between 1 week of activity and the degree of overweight/obesity in children and adolescents at a certain point in time, there is an unstable relationship, as individual obesity may be influenced by factors such as genetics and environment. Therefore, follow-up studies are needed to explore the relationship between MVPA activity and overweight/obesity, and to eliminate biases caused by individual differences, in order to reveal a stable causal relationship between the two.

This study found that the BMI of both parents significantly affects the BMI of adolescents. Overall, the BMI of parents is beneficial in suppressing underweight in adolescents. However, the BMI of fathers significantly affects the incidence of male obesity (OR 1.28, p=0.01) and female overweight (OR 1.16, p=0.01). For every unit increase in maternal BMI, the obesity rate of daughters increases by 1.21 times, but it has no effect on the obesity rate of sons. This discovery confirms previous research and this relationship may be determined by genetic factors.^47,48^ In the selection of exercise purposes, if the purpose of exercise is to be with family or friends, this will significantly increase the risk of obesity in female adolescents (OR 2.62, p=0.001). Therefore, it can be inferred that people with this purpose do not have a habit of regular exercise and this lifestyle will lead to the risk of obesity. When adolescents consider exercise as a lifestyle habit, the incidence of obesity in female adolescents significantly decreases to 0.26 times the original level. This suggests that if female adolescents can develop regular exercise habits, it will greatly help them maintain a normal body shape.

Socio-economic status has been proven to be an important determinant of health and physical activity among children and adolescents in Western countries, as parents can influence their children's physical activity behaviour by controlling their time and resource structure.^21,22^ Scholars have found in their research on adults ≥18 y of age that the higher the socio-economic status, the greater their demand for exercise and the higher the frequency of spontaneous physical activity.^49^ However, some scholars have found^24^ that adolescents from families with low socio-economic status tend to engage in more physical activities. This is because families with high socio-economic status also have higher social capital and are more likely to have high expectations for their children, hoping that they can enter higher-ranked schools. Therefore, they tend to convert their economic capital into tutoring, resulting in a significantly higher proportion of their children participating in subject tutoring. This may reduce the time that teenagers can engage in physical activities. This study found that the work status of parents (full-time work) has a positive impact on the BMI of female adolescents, which is consistent with the conclusions of pre-selection research.^50^ On the other hand, monthly household income has a significant negative impact on the BMI of male and female adolescents, indicating that the better the economic background of female adolescents, the more advantageous it is in controlling their BMI values, which is consistent with many previous research results.^51–53^

In summary, during the growth process of adolescents, their BMI is influenced by the interaction of biological and environmental factors, among which environmental factors are more complex and varied, including macro systems, external systems, intermediary systems and micro systems. The macro system refers to the influence of social and cultural factors, political factors, legal issues, social classes and current events around the world on the physical activity behaviour of children and adolescents. External systems refer to factors that, although not directly involved, still affect the physical activities of children and adolescents during their growth, such as the working environment of parents, the direction of school education and the use of community resources. The connections and relationships between families, schools, friends and communities of children and adolescents are within the scope of the intermediary system. The microsystem refers to the living environment, including the relationship between children and families and the connection and communication between schools and extracurricular activities. Therefore, exploring the influencing factors of adolescent BMI should have a global perspective. Scholars have confirmed that there is a reverse causal relationship between adolescent exercise duration and BMI, which may lead to endogeneity issues and indicate that individuals may become aware of being overweight or obese and engage in more physical activity.^54^ After correcting for endogeneity issues, there is a significant negative effect of physical activity levels on the likelihood of obesity.^55^ When analysing the impact of physical activity levels on adolescent BMI, this study did not consider endogeneity issues. Perhaps future research directions can delve deeper into endogeneity issues. In addition, obesity in adolescents is closely related to their sedentary lifestyle. However, due to the current lack of standardized and clear definitions of sedentary behaviour, and the absence of a significant negative relationship between sedentary time and physical activity time, in-depth analysis of the impact of sedentary behaviour on adolescent BMI is needed and should comprehensively consider relevant data such as adolescent dietary habits.

### Limitations

Like most other studies, the results of this study are limited by cross-sectional data factors and cannot control for potential factors affecting obesity. Long-term tracking of adolescents is needed in the future to obtain more accurate results. This study used the IPAQ to evaluate the physical activity level of adolescents, and this subjective method may lead to overestimation or underestimation of the results. In the future, objective measurement methods will be needed to make more accurate evaluations. This study did not consider endogeneity or dietary behaviour issues among adolescents. In the future, endogeneity issues should be included and sedentary lifestyles and dietary habits among adolescents should be included in order to comprehensively analyse the causes and prevention of adolescent obesity.

## Conclusions

Adequate exercise is beneficial for both obese and underweight male and female adolescents. It can help obese male and female adolescents lose weight and can also help underweight adolescents improve their BMI. As age increases, the impact of BMI on males is greater than that on females, while fathers have a greater impact on the BMI of boys than girls, while mothers have the opposite effect. The higher the family income, the greater the likelihood of girls becoming obese, and full-time working mothers may increase the risk of adolescent obesity. The purpose of exercise significantly affects the BMI of adolescents. If male and female adolescents use physical exercise as a lifestyle habit, it will significantly improve their physical condition.

## Data Availability

The datasets of the current study are available from the corresponding author upon reasonable request.
